# Diltiazem Prophylaxis for the Prevention of Atrial Fibrillation in Patients Undergoing Thoracoabdominal Esophagectomy: A Retrospective Cohort Study

**DOI:** 10.1007/s00268-020-05444-y

**Published:** 2020-03-04

**Authors:** Marcel Hochreiter, Thomas Schmidt, Benedikt H. Siegler, Leila Sisic, Karsten Schmidt, Thomas Bruckner, Beat P. Müller-Stich, Markus K. Diener, Markus A. Weigand, Markus W. Büchler, Cornelius J. Busch

**Affiliations:** 1 Department of Anesthesiology and Intensive Care Medicine, Essen University Hospital, Essen, Germany; 2 Department of Anesthesiology and Intensive Care Medicine, Heidelberg University Hospital, Heidelberg, Germany; 3 Department of General, Visceral and Transplantation Surgery, Heidelberg University Hospital, Heidelberg, Germany; 4 Institute of Medical Biometry and Informatics, Heidelberg University Hospital, Heidelberg, Germany

## Abstract

**Background:**

Atrial fibrillation (AF) represents the most frequent arrhythmic disorder after thoracoabdominal esophageal resection and is associated with a significant increase in perioperative morbidity and mortality.

**Methods:**

In this retrospective cohort study, 167 patients who underwent thoracoabdominal esophagectomy at a large university hospital were assessed. We compared patients who received a 14-day postoperative course of diltiazem with a control group of patients who did not undergo diltiazem prophylaxis. Diltiazem therapy started immediately upon admission to the intensive care unit (ICU) with a loading dose of 0.25 mg/kg bodyweight (i.v.) followed by continuous infusion (0.1 mg/kg bodyweight/h) for 40–48 h. Oral administration (Dilzem^®^ 180 mg uno retard, once a day) was started on postoperative day 3.

**Results:**

A total of 117 patients were assessed. Twelve (10.3%) of all patients developed postoperative new-onset atrial fibrillation in the first 30 days after surgical intervention. Prevalence of new-onset AF showed no significant differences between the diltiazem group and control group (*p* = 0.74). The prevalence of bradycardia (14.7% vs. 3.6%; *p* = 0.03) and dose of norepinephrine required (0.09 vs. 0.04 µg/kg bodyweight/min; *p* = 0.04) were higher in the diltiazem group. There were no significant differences between the groups for the median postoperative duration of hospital/ICU stay or mortality.

**Conclusions:**

A prophylactic 14-day postoperative course of diltiazem was not associated with a reduction in new-onset AF or 30-day mortality following thoracoabdominal esophagectomy. Prophylactic diltiazem therapy was associated with drug-related adverse effects such as bradycardia and increased requirement of norepinephrine.

German Clinical Trial Registration Number: DKRS00016631.

## Introduction

Postoperative atrial fibrillation (POAF) is the most common cardiac arrhythmia after esophagectomy and has been reported to appear in ≤44% of patients [1, 2]. Although POAF in thoracic surgery has been considered to be temporary in most cases [3], its occurrence in the postoperative phase is associated with a substantial increase in morbidity, mortality, resource utilization and long-term risk of stroke [2, 4–6]. Direct results of POAF include a decrease in cardiac output and development of atrial thrombosis. The etiology of POAF is incompletely understood.

Development of POAF requires vulnerable atrial tissue and a trigger to initiate AF [7]. The risk factors for POAF are being male, age >75 years, nicotine/alcohol consumption, cardiopulmonary comorbidities (i.e., arterial hypertension, chronic obstructive pulmonary disease, congestive heart failure), preoperative episode of AF, as well as the location (irritation of atria) and magnitude of surgery (including esophagectomy) [8–10]. Surgical procedures are associated with local or systemic inflammation affecting the vulnerability of the atrial substrate to POAF [11]. Usually, AF onset occurs on postoperative day (POD)2 and POD3 [12], whereas the risk of arrhythmias decreases over the first postoperative month to that before surgery independent of the treatment given [13].

POAF represents a major (but potentially preventable) adverse outcome. For thoracic surgical procedures, current recommendations from the American Association for Thoracic Surgery Taskforce favor prevention strategies based on pharmacology [7]. In detail: (1) oral beta-blockers should be continued after surgery to avoid withdrawal; (2) all patients should receive magnesium (i.v.) perioperatively if the serum magnesium level is low; (3) for most patients at an increased risk of POAF development, preventive administration of diltiazem or amiodarone (especially for esophagectomy) may be reasonable [7]. Undoubtedly, amiodarone is among the most efficacious antiarrhythmic agents, but its use is associated with potentially serious toxicity, so in our institution diltiazem is used for patients with preserved cardiac function for PAOF prevention [14].

Based on several double-blinded controlled trials in cardiac surgery and lung surgery, the efficacy of calcium channel blockers has been tested for the prophylaxis of POAF [15–17]. In those studies, the prevalence of POAF was reduced by ~50% [18]. Diltiazem use is associated with a far lower prevalence of hypotension than verapamil use, so diltiazem is the recommended calcium blocking agent. Diltiazem inhibits L-type calcium channels in vascular and conduction tissue, and especially in nodal tissue [19].

Despite the demonstrable prophylactic effect of diltiazem against POAF in cardiac surgery and lung surgery, data regarding its efficacy in the prevention of POAF in patients undergoing esophagectomy are lacking. Therefore, we evaluated the effect of 14-day postoperative prophylaxis with the calcium antagonist diltiazem to prevent POAF in patients undergoing thoracoabdominal esophagectomy.

## Patients and methods

### Ethical approval of the study protocol

The present study was undertaken in accordance with the 1975 Helsinki Declaration and its later amendments and after approval from the ethics committee of the Medical Faculty of Heidelberg University (Heidelberg, Germany; S-493/2016). Due to its retrospective character, written informed consent was not needed.

### Data collection

A retrospective cohort study was conducted to evaluate the efficacy and safety of postoperative administration of diltiazem in patients undergoing thoracoabdominal esophagectomy at Heidelberg University Hospital. Patients were identified from an internal clinical prospectively maintained database. Electronic medical records were reviewed for demographic and clinical characteristics, concomitant diseases, medications, surgery type, laboratory values, occurrence of POAF, as well as the prevalence and nature of postoperative adverse events and complications.

### Outcome measures

The primary outcome measure was the prevalence of new-onset AF lasting ≥30 s or for the duration of the electrocardiography recording (if <30 s) within 30 days after thoracoabdominal esophagectomy. Secondary outcome measures were duration of postoperative stay in hospital or intensive care unit (ICU), prevalence of adverse events, as well as 30-day and 90-day mortality. Bradycardia was defined as heart rate <50 beats per minute. Hypotension was defined as systolic blood pressure <90 mmHg that necessitated administration of fluid/and or vasopressors.

### Study population and surgical procedure

**Fig. 1:**
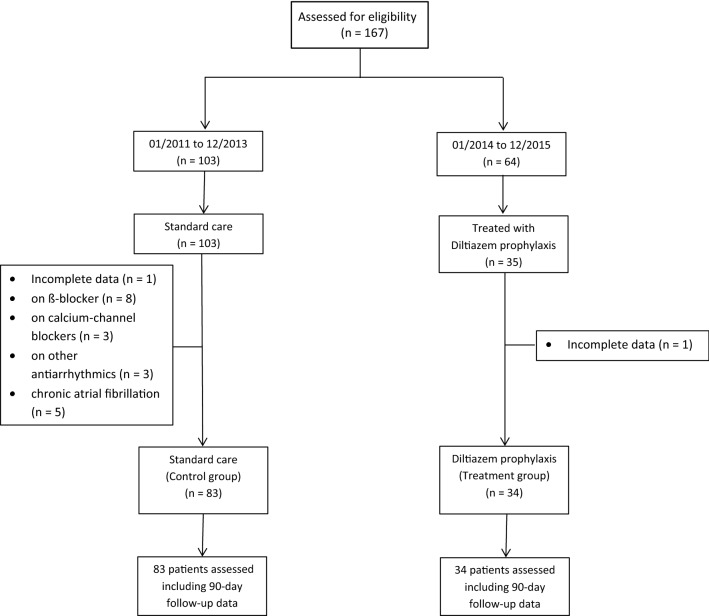
Flowchart of the study enrollment population

All patients aged ≥18 years who underwent thoracoabdominal esophagectomy with abdominal and mediastinal lymphadenectomy for adenocarcinoma or squamous cell carcinoma between 2011 and 2015 were included in data assessment and screened for eligibility from a prospectively maintained database. Thoracoabdominal esophagectomy was undertaken *via* laparotomy followed by right-sided thoracotomy and intrathoracic anastomosis (Ivor Lewis procedure) [20]. Patients with incomplete data sets, disturbances of sinus functions, second- and third-degree atrioventricular blocks, who were taking antiarrhythmic drugs preoperatively or with contraindications against diltiazem prophylaxis (i.e., medication with β-receptor antagonists, reduced cardiac function) were excluded from the final analysis (Fig. 1).

### Perioperative care and postoperative diltiazem prophylaxis

After surgery, patients were admitted routinely to the ICU. In accordance with evidence for prevention of AF related to general thoracic surgery [7], from 2014 onwards diltiazem prophylaxis was conducted routinely in patients undergoing thoracoabdominal esophagectomy. In accordance with the local ICU protocol, contraindications for diltiazem were reduced cardiac function, disturbances of sinus functions, second- and third-degree atrioventricular blocks, or treatment with antiarrhythmic drugs before surgery.

Accordingly, patients included in the present study were assigned to the control group (before 2014) or treatment group (after 2014). Patients in both study groups were treated identically with regard to surgical procedure, perioperative administration of fluids, enteral feeding, and drainage management. In accordance with the local ICU protocol: (1) the serum levels of potassium ions (K^+^) were maintained at the high end of normal (4.5–5.5 mEq/L); (2) in the event of clinical deterioration computed tomography was undertaken (including evidence of infection).

In the treatment group, diltiazem prophylaxis was given as per the local ICU protocol for 14 days. Therapy started immediately upon ICU admission with a loading dose of diltiazem (0.25 mg/kg bodyweight, i.v.) given over 30 min, followed by continuous infusion (0.1 mg/kg bodyweight/h) for 40–48 h. Administration was discontinued if the pulse or systolic blood pressure dropped below 60 beats per min or 90 mmHg, respectively. Oral administration (Dilzem^®^ 180 uno retard, once a day) started at POD3 when patients were relocated to the normal ward. All patients underwent continuous telemetry monitoring until POD14. After POD14, vital signs were documented three times a day until the patient was discharged from hospital, and a 12-lead ECG was taken (if appropriate).

### Statistical analyses

Descriptive statistics for empirical distributions are the mean ± SD (continuous data) and absolute and relative frequencies (categorical data). Possible differences between patient groups were evaluated using the Mann–Whitney *U*-test for continuous variables and Chi-square test or Fisher’s exact test (where appropriate) for categorical variables. The 95% confidence interval (CI) of the difference was calculated. *p* < 0.05 was considered significant. Analyses were performed using SASTM 9.4 (SAS Institute, Cary, NC, USA).

## Results

### Patient characteristics

In total, between 2011 and 2015, 167 patients underwent thoracoabdominal esophagectomy at our institution. Fifty patients were excluded from the final data analysis, mainly because of chronic AF or because they were already on ß-blockers or calcium channel blockers as background therapy (Fig. 1). Of the remaining 117 patients, 34 individuals (29%) received diltiazem prophylaxis, whereas 83 (71%) did not. In both groups, the duration of follow-up was 90 days. Both groups were comparable in terms of age, sex, body mass index, underlying disease and preoperative treatment. Table 1 summarizes the baseline characteristics of all included patients.

**Table 1:** Characteristics of the study cohort

	Diltiazem (*n* = 34)	Control (*n* = 83)	*p*
Age (years)	60.0 (±9.8)	60.9 (±11.8)	0.188
Sex			0.105
Male	30 (88.2)	62 (74.7)	
Female	4 (11.8)	21 (25.3)	
BMI (kg/m^2^)	23.8 (±3.9)	25.2 (±4.6)	0.124
Diabetes mellitus	4 (11.8)	4 (4.8)	0.177
Arterial hypertension	14 (41.2)	28 (33.7)	0.446
Coronary heart disease	2 (5.9)	8 (9.6)	0.509
COPD	4 (11.8)	11 (13.2)	0.827
Renal insufficiency	1 (2.9)	1 (1.2)	0.511
Preoperative chemotherapy and/or radiotherapy	23 (67.6)	68 (81.9)	0.092

Data are the mean ± standard deviation or number (%)

*BMI* body mass index, *COPD* chronic obstructive pulmonary disease

### Perioperative management and surgical outcome

Data for intraoperative management and postoperative outcome are listed in Tables 2 and 3, respectively. Most patients received combined epidural and general anesthesia (control group, 81.9%; treatment group, 91.2%; *p* = 0.21). The duration of the surgical procedure, blood loss, as well as the intraoperative volume of fluids and transfused blood, were comparable between the groups. One patient in the treatment group (2.9%) and two patients in the control group (2.4%) needed continuous veno-venous hemofiltration after surgery (*p* = 0.87). There was no significant difference between the groups in the prevalence of postoperative complications, such as sepsis (*p* = 0.40), anastomotic leakage (*p* = 0.41), pneumonia (*p* = 0.50) or requirement of respiratory support (*p* = 0.95). No POAF patients were diagnosed with cardioembolic stroke during hospital stay.

**Table 2:** Intraoperative data

	Diltiazem (*n* = 34)	Control (*n* = 83)	Difference (95% CI)	*p*
Duration of surgery (min)	266 (±62)	259 (±110)	7.0 (−25.1 to 39.1)	0.728
Intraoperative fluid administration (mL)	3610 (±1028)	3610 (±1375)	0.1 (−462 to 462.0)	>0.999
Blood loss (mL)	763 (±382)	832 (±514)	−69 (−241 to 103)	0.483
Units of blood transfused	0.41 (±1.08)	0.72 (±1.60)	−0.31 (−0.82 to 0.20)	0.301
Need for a thoracic epidural catheter	31 (91.2)	68 (81.9)	9.3% (−7.1 to 21.4)	0.208

Data are the mean ± standard deviation or number (%)

*CI* confidence interval

**Table 3:** Postoperative outcome

	Diltiazem (*n* = 34)	Control (*n* = 83)	Difference (95% CI)	*p*
*Cardiovascular system*				
Hypotension*	14 (41.2)	31 (37.4)	3.8% (−15.3 to 24.1)	0.699
Bradycardia**	5 (14.7)	3 (3.6)	11.1% (−27.5 to 0.0)	0.031
Need for catecholamines	19 (55.9)	32 (38.6)	17.3% (−3.5 to 36.9)	0.086
Norepinephrin (µg/kg/min)	0.09 (±0.13)	0.04 (±0.07)	0.05 (0.00 to 0.10)	0.041
Dobutamin (µg/kg/min)	0.76 (±1.39)	0.76 (±1.88)	−0.05 (−0.68 to 0.59)	0.888
Atrial fibrillation (new onset)	3 (8.8)	9 (10.8)	−2.0% (−13.1 to 13.8)	0.744
Atrial fibrillation (new onset) as a solitary complication	1 (2.9)	3 (3.6)	−0.7% (−8.2 to 12.2)	0.856
*Respiratory system*				
Duration of respiratory support in the ICU (h)***	24.9 (±80.4)	26.2 (±100.0)	−1.3 (−37.2 to 34.5)	0.946
Pneumonia	3 (8.8)	11 (13.2)	−4.4% (−16.0 to 11.5)	0.503
*Other*				
CVVH	1 (2.9)	2 (2.4)	0.5% (−6.3 to 13.5)	0.869
Sepsis	2 (5.9)	9 (10.8)	−4.9% (−15.3 to 9.7)	0.404
Anastomotic leakage	3 (8.8)	12 (14.4)	−5.6% (−17.4 to 10.3)	0.408
*Mortality*				
30 days	3 (8.8)	4 (4.8)	4.0% (−5.5 to 19.4)	0.407
90 days	3 (8.8)	5 (6.0)	2.8% (−7.1 to 18.5)	0.586

Data are the mean ± standard deviation or number (%)

*CI* confidence interval, *ICU* intensive care unit, *CVVH* continuous veno-venous hemofiltration

*Systolic pressure <90 mmHg, **heart rate <50 beats/min, ***ventilation and noninvasive ventilation

### Postoperative prevalence of AF and need for cardio-circulatory medication

Overall, POAF occurred in 12 patients after esophagectomy (10.3%) (Table 3). Most patients (10/12, 83.3%) developed POAF within 72 h of surgery. The proportion of patients with POAF did not differ significantly between the control group and individuals with postoperative diltiazem prophylaxis (difference −2%; 95% CI −13.1 to 13.8; *p* = 0.74). No significant differences were found in the number of patients requiring postoperative administration of catecholamines (*p* = 0.09), including dobutamine (*p* = 0.89). Patients in the diltiazem group received postoperative higher doses of norepinephrine (0.09 vs. 0.04 µg/kg bodyweight/min, *p* = 0.04) in the ICU (Table 3). The prevalence of bradycardia was higher in the diltiazem group than in the control group (*p* = 0.03). Five (14.7%) of diltiazem-treated patients required temporary discontinuation of the drug because of bradycardia and hypotension.

POAF was frequently associated with one or more other complications. The most common surgical complication associated with POAF was anastomotic leakage. POAF and anastomotic leakage occurred in 8 of 12 patients. Four of them had additional pulmonary complications. POAF presented as a solitary complication in 4 of 12 patients. It took on average 3 days after the onset of POAF to diagnose anastomotic leakage, whereas pulmonary complications preceded the onset of POAF. Neither 30-day nor 90-day mortality differed significantly between the control group and diltiazem group (*p* = 0.41 and *p* = 0.59) (Table 3).

### Duration of postoperative stay in hospital or ICU

The median postoperative duration of hospital stay and ICU stay in the overall study cohort (*n* = 117) was 15 days (interquartile range [IQR], 13–20 days) and 2 (IQR, 1–4 days), respectively. There was no significant difference in the median duration of postoperative hospital stay in the diltiazem group compared with that in the control group (14 days, IQR 12–19 vs. 15 days, IQR 13–21 days; *p* = 0.15) nor in the median duration of ICU stay between the two groups (2 days, IQR 1–5 vs. 2 days, IQR 1–4 days, *p* = 0.35).

## Discussion

Esophagus resection through a thoracoabdominal approach is the first-line treatment for esophagus carcinoma [21, 22]. Besides surgical complications [23] and pulmonary infection [24], AF is one of the most common complications and is associated with a substantial increase in morbidity, mortality, resource utilization and long-term risk of stroke. Reducing the prevalence of POAF in esophagectomy is extremely important.

We showed that postoperative antiarrhythmic prophylaxis with diltiazem for 14 days did not reduce the prevalence of new-onset AF in the first 30 days after surgery and did not influence mortality after thoracoabdominal esophagectomy.

Patients who have a thoracoabdominal esophagectomy are at a high risk of developing new-onset AF, and a range from 10 to 44% has been reported [8, 25, 26]. The overall prevalence of POAF observed in the present study was 10.3%, which was lower than expected. However, it is difficult to compare data between studies without taking risk factors into account. A consistent clinical predictor of POAF after major thoracic procedures is older age [12, 26]. Aging causes degenerative changes in atrial anatomy, including dilation and fibrosis, which results in a vulnerable atrial myocardium [27]. This is accompanied by shorter atrial effective refractoriness, longer sinoatrial and nodal conduction times, atrial stiffening and splitting of the atrial excitation waveform caused by the pectinated trabeculae [15]. Lohani et al. [28] showed that patients aged >65 years experienced a higher prevalence of POAF after esophagectomy. In the present study, the mean age was 60.4 years, which might have lowered the risk of developing POAF.

A further important risk factor for POAF is the degree of surgical stress, which causes systemic and local inflammation. Okamura et al. [29] showed that, in patients after esophagectomy, the longer the surgical procedure, the higher the release of proinflammatory cytokines in serum (e.g., interleukin (IL)-6 and IL-8). Subsequently, a meta-analysis demonstrated that an increase in circulating levels of proinflammatory factors was associated with a greater risk of POAF in the general population as well as in patients who underwent coronary artery bypass grafting [30]. The median duration of surgery in the present study was 261 min, which is shorter compared with the data from a study by Tisdale et al. [31], who reported a median duration of surgery of 477.5 min and prevalence of POAF of 40%. However, some cases of subclinical and transient POAF may have been missed because of less intensive cardiac monitoring on the normal ward after POD14 during hospitalization.

The high prevalence of POAF after major thoracic surgery has led to several studies investigating the role of postoperative pharmacologic prophylaxis [16, 31, 32]. However, much of the evidence has been extrapolated from cardiac surgery studies. Only a few trials have been conducted exclusively in patients undergoing esophagectomy [25, 33, 34]. Tisdale et al. evaluated, in one randomized controlled study (*n* = 80) and very recently in a retrospective cohort study (*n* = 220), the effect of amiodarone on POAF with esophagectomy and found fewer patients with POAF in the amiodarone group. The randomized controlled study did not find a difference in other parameters between the two groups, but the cohort study revealed that amiodarone (i.v.) was associated with hypotension, bradycardia and corrected prolongation of the QT interval [25, 34]. However, due to potential side effects such as lung toxicity (probably only in high-dose therapy), amiodarone is not used widely in clinical practice for this indication. Ojima et al. investigated landiolol use in patients after esophagectomy. AF occurred in 30% of cases, whereas perioperative β-blockade reduced it to 10% [33]. Prophylactic use of ß-blockers in the perioperative setting is controversial. Studies in noncardiac thoracic surgery have shown a high prevalence of hypotension and bradycardia [32, 35]. In 2014, guidelines set by the American Association for Thoracic Surgery did not recommend prophylactic use of ß-blockers for POAF prevention unless the patient was already on ß-blocker therapy before surgery to avoid withdrawal [7].

Previously, prophylactic use of diltiazem showed promising results in reducing POAF after lung surgery. In two prospective randomized trials, Amar et al. reported significant reduction in POAF compared with that elicited by placebo or digoxin [15, 36]. We used the same dose regimen in patients after esophagus resection. Interestingly, our data showed a similar prevalence of POAF in patients with diltiazem prophylaxis compared with those receiving standard care. More side effects (e.g., hypotension, bradycardia) were found in patients receiving diltiazem. Subsequent administration of diltiazem was associated with significantly higher doses of norepinephrine. This strategy is potentially harmful because during esophagectomy, multiple arteries are ligated and the newly formed gastric tube is dependent only on the right gastro-epiploic artery. Fumagalli et al. [37] showed in a observational study that hypotensive episodes (decrease in systolic pressure >30% of the baseline value for >5 min) and use of vasopressors worsened local perfusion and were the main contributing factors for anastomotic leaks in patients undergoing esophagectomy. However, our data showed a similar prevalence of anastomotic leaks in both groups.

Bradycardia is a common side effect of diltiazem treatment due to inhibition of L-type calcium channels in sinus nodes. This negative chronotropic effect was also observed in the diltiazem group of the present study. These data are in accordance with results from other studies because patients with ischemic heart disease showed lower heart rates starting at 0.25 mg/kg (i.v.) [38, 39], which is equivalent to the initial loading dose. Another possible mechanism of bradycardia might be thoracic epidural anesthesia *via* blockade of cardiac rami [40], but this was not evident in our data.

Embolic stroke is the most feared complication of POAF. Gialdini et al. evaluated stroke risk due to POAF from a database including >1 million noncardiac surgical patients. They documented a 1.47% cumulative risk of stroke 1 year after hospitalization, compared with 0.36% in patients who did not develop POAF [6]. Anticoagulation is the cornerstone of prevention of embolic stoke, but it is challenging in the perioperative period. Postoperative patients carry a higher risk of bleeding and a hypercoagulable state simultaneously. American and European guidelines state that it is reasonable to administer antithrombotic medication if POAF persists for >48 h, but this is not a specific recommendation for noncardiac surgery patients because direct evidence is not available [41, 42].

We demonstrated that anastomotic leakage was a frequent morbidity in patients with POAF. Eight out of 12 patients (66.7%) with POAF showed anastomotic leakage. This value is consistent with a recent study by Sessing et al. [43] in which 77.4% of patients with POAF after esophagectomy had infectious complications such as pneumonia (41.9%) and anastomotic leakage or conduit necrosis (43.0%). These data underline the hypothesis that POAF is frequently associated with postoperative complications and is likely a systemic manifestation of a local complication. The median time interval between the onset of POAF and diagnosis of anastomotic leakage was 3 days. Hence, POAF might trigger the medical team to carry out an early investigation to diagnose infectious complications. Diltiazem prophylaxis might not prevent POAF in all cases because a different grade of systemic inflammation in infectious complications potentially overrules the antiarrhythmic effect of diltiazem. In contrast, successful pharmacologic prophylaxis for POAF in esophagectomy might mask a warning sign and subsequently delay the diagnosis and treatment of anastomotic leaks. However, this effect was not evident in our data.

Overall, preventing POAF with a single drug that affects a single pathway seems unlikely because the etiology of POAF is multifactorial and its mechanism of action is incompletely understood. To prevent POAF in clinical practice, the focus should be on control of preventable perioperative risk factors: electrolyte imbalances, perioperative hypervolemia/hypovolemia, hypotension and anemia [44]. Moreover, we revealed that most episodes of POAF arose in the setting of anastomotic and septic complications. Consequently, an optimal technical outcome is important. Technical improvements like ischemic conditioning of the gastric conduit before esophagectomy [45] as well as intraoperative indocyanine fluorescence are promising and have been reported to improve tissue perfusion. The latter still needs randomized, multicenter trials to proof the benefit [46]. Additionally, minimal invasive procedures might reduce the perioperative trauma. Still, these improvements are limited by the anatomy of the patient and up to now anastomotic leakage remains unchanged. From the technical point of view, it would be conceivable that the site of anastomosis matters. There are data that cervical and thoracic anastomosis are equally safe [47, 48], but there are also data that intrathoracical anastomosis leads to less leakage and wound infection [49]. Promising results have been reported of enhanced recovery protocols for esophagectomy. A systematic review and pooled analyses showed a reduced incidence of anastomotic leak for patients followed by an enhanced recovery protocol compared to those undergoing esophagectomy followed by a usual care [50]. Alternative surgical approaches combining laparoscopy with thoracotomy or, if feasible, a completely minimally invasive procedure lead to reduce pulmonary complications but did not change the number of anastomotic leakage [51].

In case of a suspected leakage, early detection of infectious complications by clinical examination, laboratory studies to screen for signs of infection (e.g., increased leukocyte count, procalcitonin level or CRP level) and subsequent CT of the chest and abdomen and/or endoscopy (if appropriate) is necessary.

Our study had several limitations. It was a single-center, retrospective analysis, and there was a selection bias because many patients with thoracoabdominal esophagectomy were excluded. An advantage of our design was the comparability of study participants with regard to perioperative regimens. All data were derived from an in-patient routine database of our hospital and were not collected specifically for our study. Only documented cases of POAF were taken into account, which may have underestimated the true prevalence of POAF. Furthermore, patients may not have reported outpatient episodes of AF to their general practitioner. Hence, these outcomes were missing and would have led to incorrect estimation of the prevalence of POAF in this population. Stawicki et al. [52] reported the onset of POAF to be ~90% within POD3 after esophagectomy. Thus, the number of missed POAFs after hospital discharge is probably low.

## Conclusions

In this retrospective analysis, a prophylactic 14-day postoperative course of diltiazem was not associated with a reduction in the prevalence of new-onset AF or 30-day mortality following thoracoabdominal esophagectomy. Prophylactic diltiazem was associated with drug-related adverse effects such as bradycardia and increased requirement of norepinephrine. Consequently, the prophylactic effect of diltiazem is questionable, and intensivists should carefully weigh the risk versus theoretical benefit of diltiazem in patients after thoracoabdominal esophagectomy. POAF seldom occurs without complications, so it could function as an early warning sign for anastomotic leaks and may, thus, be of clinical importance.

Based on the results of this study, we no longer use diltiazem prophylaxis as standard care for these patients. Instead, we focus on control of preventable perioperative risk factors and introduced an enhanced recovery protocol.
